# Nursing Research, CER, PICO and PCORI

**DOI:** 10.4172/2471-9846.1000206

**Published:** 2017-12-31

**Authors:** Darpan I Patel

**Affiliations:** School of Nursing, The University of Texas Health Science Center at San Antonio, San Antonio, TX, USA

**Keywords:** Patient centered outcomes research, Patient engagement, Healthcare systems, Community engagement

## Abstract

Community and public health nurse researchers encompass a unique cohort of nurse researchers that have the skills and capacity to lead projects and programs of science centered on improvement of patient outcomes through methods of comparative effectiveness research (CER). CER, as a general method, has been taught to all nurses in the form of the PICO question to improve evidence-based practices. As the climate for funding becomes more and more competitive, nurse researchers are primed to lead the change in improving patient outcomes through patient centered outcomes research (PCOR). However, the number of projects funded by agencies like the Patient Centered Outcomes Research Institute, fall well below the capabilities of the field. The purpose of this commentary is to promote the field of PCOR and encourage novice and experienced nurse researchers to apply for funding from the PCORI by introducing different methods for building capacity and promoting engagement in the national conversations of PCOR and CER.

## Introduction

Academic nurses and nurse researchers have been trained starting with their undergraduate education on the development of evidence-based practices to ensure that care that is received by their patients is safe, timely, effective, efficient, equitable and patient centered [1]. There is an urgent need for healthcare transformation through quality improvement and patient safety initiatives [2]. Nurses represent an important aspect of this transformation and bring a unique perspective of patient-centeredness and systems thinking. Nursing research encompasses a wide scope of scientific inquiry including clinical research, health systems research and outcomes-based research [3, 4]. However, the recent drive to engage patients and stakeholders in complex research programs calls for a transition in the nursing research paradigm with an emphasis on patient engagement [5]. Continued success in nursing research is at risk due to a lack of nurseled programs of research incorporating patient centered outcomes research (PCOR). With nurses at the center for patient care, the need to increase capacity of nurse scientists to conduct PCOR is essential. The purpose of this commentary is to promote the field of PCOR and encourage novice and experienced nurse researchers to apply for funding from the Patient Centered Outcomes Research Institute (PCORI) by introducing different methods for building capacity and promoting engagement in the national conversations of PCOR and comparative effectiveness research (CER).

## PCORI

The Patient Centered Outcomes Research Institute was created was created by congress in 2010 to assist patients, clinicians and others in making informed decisions by improving the quality and relevance of evidence based practices for preventing, diagnosing, treating, monitoring and managing health problems [5]. PCORI’s mission is to help people make informed healthcare decisions and improve healthcare delivery and outcomes, by producing and promoting high-integrity, evidence-based information that comes from research guided by patients, caregivers and the broader healthcare community [5]. PCORI funds comparative effectiveness research (CER) to evaluate and compare two or more practices to identify the most effective tools, strategies, pharmaceuticals, practice and other strategies used in the prevention, treatment, diagnosis and management of illness in individuals susceptible or currently living with the condition [6].

Patient-centered outcomes research (PCOR) is CER that helps answer the following questions that a patient is likely to pose [6]:

- “Given my personal characteristics, conditions and preferences, what should I expect will happen to me?”
- “What are my options and what are the benefits and harms of those options?”
- “What can I do to improve the outcomes that are most important to me?”
- “How can the health care system improve my chances of achieving the outcomes I prefer?”

## Nursing Research and PCOR

Nurse researchers are primed to be successful in receiving PCORI funding due to their training on comparative effectiveness research as it chance to relates to evidence-based practice. However, for some reason or another, the number of nurse led projects funded by PCORI does not mirror the capabilities of nurse researchers in this arena. In a search of the PCORI funded projects database, less than 50 projects of the over 1,200 projects funded by PCORI are led by a Nurse Principal Investigator, accounting for only 4% of all funded projects. There are multiple reasons as to why these low numbers may be the case. Perhaps it is grant writing ability, lack of knowledge of the funding mechanisms, lack of training, lack of time, lack of patient partners that are required for PCORI funding or some combination of these that makes it difficult for nurse researchers to be successful in securing project funding from PCORI. However, given the background of community engagement and public health focus that majority of nurses have, there exists an opportunity for more nurse researchers to be successful in securing PCORI funding. To address these issues, there exists a number of venues where new and established nurse researchers can boost their capacity for being successful in conducting PCOR. Conferences like the annual PCORI conference held in Washington DC or the annual conference for Community Engagement and Healthcare Improvement hosted by the School of Nursing at UT Health San Antonio offer opportunities for nurse researchers to build capacity on PCOR methods and patient engagement strategies [3].

Funding opportunities also exist for individuals and groups that are looking to develop a PCOR-centered program [6]. The Eugene Washington PCORI Engagement Award and the Pipeline to Proposal funding mechanism at PCORI provide an opportunity for research teams to be developed with community partners to address topical issues that are of concern to community stakeholders [5]

Advocacy of nursing’s role in PCOR can also be done through the Ambassador program [7]. The role of the PCORI Ambassador is to build positive relationships within the healthcare community and work in partnership with other Ambassadors and healthcare professionals to promote research that incorporates the perspectives of patients and other stakeholders, including nurses [7]. By becoming an Ambassador, nurse researchers will be able to participate in PCOR training to enhance readiness for PCORI-funded research and be active partners in the wide national network of PCOR [7].

## PCORI, CER and Nursing Research

The type of research capable of being produced by nurse researchers will have significant impact to evidence-based practice, education and future research associated with improving patient outcomes. Nurse researchers must understand the climate in which funding is being made and not feel intimidated by the funding arena. Nurses are inherently prepared to conduct CER and PCOR given the foundation of nursing practice built on developing evidence-based practices through generating PICO questions. In the end, PICO is CER and nurse researchers are primed to join and be leaders in this movement to improve patient outcomes through patient engaged research.
